# Do male and female heads of households have different beliefs about gender equity among young people in Nigeria?

**DOI:** 10.3389/fsoc.2024.1354991

**Published:** 2024-08-14

**Authors:** Ozioma Patricia Nwankpa, Chinazom N. Ekwueme, Ifeyinwa Akamike, Chinyere Ojiugo Mbachu, Obinna Onwujekwe

**Affiliations:** ^1^Health Policy Research Group, University of Nigeria, Enugu Campus, Enugu, Nigeria; ^2^Department of Community Medicine, Alex Ekwueme Federal University Teaching Hospital, Abakaliki, Abakaliki, Nigeria

**Keywords:** gender equity, female household heads, male household heads, Nigeria, young people, rights, sexual and reproductive health

## Abstract

**Introduction:**

Gender-transformative approaches (GTAs) have been successfully carried out to address harmful gender norms and power imbalances to promote more gender equitability. However, to improve the health and wellbeing of young people, it is necessary to involve household heads by positively transforming their beliefs on gender equity and norms.

**Methods:**

This study was cross-sectional quantitative research undertaken in six local government areas in Ebonyi State, Nigeria. The study population consisted of household heads in households with young people aged 15–24 years. Data were collected for 15 days using paper and electronic copies of the questionnaire. Descriptive, bivariate, and logistic regression analyses were performed using Stata.

**Results:**

The results showed that 46.32% of male and 62.81% of female heads of households disagreed with the statement “a good woman never questions her husband’s opinions, even if she is not sure she agrees with them.” Female heads of households aged 50 years and below with an odds ratio of 0.47 (*p*-value = 0.02) suggest they were 0.47 times more likely to have a positive attitude toward the rights and privileges of young girls. Male heads of households aged 50 years and below with an odds ratio of 1.05 (*p*-value = 0.84) suggest that they were 1.05 times more likely to have a positive attitude toward the rights and privileges of young girls.

**Conclusion:**

This paper provides new knowledge on the gender norm attitude of male and female heads of households on the rights, privileges, and equity promotion of young boys and young girls, as well as its associated factors.

## Introduction

Gender norms are rules that govern beliefs on how individuals in homes, communities, or institutions should behave (Pearse and Connell, 2015; World Health Organization, 2018; Heise et al., 2019). They shape the life prospects of an individual and have significant implications for both girls and boys. These norms begin in the family by parents and are reinforced by teachers, faith leaders, peers, and exposure to media (Patel et al., 2021).

Gender norms significantly influence the health of young people, as they face diverse expectations within both their homes and society based on their gender (Buller et al., 2016; Basu et al., 2017). These expectations start early and strongly shape their attitudes, opportunities, experiences, and behaviors with significant health consequences (Closson et al., 2023), such as child marriage, unwanted pregnancy, sexually transmitted infection, exposure to domestic violence, intimate partner violence, and depression (Blum et al., 2017).

Gender-transformative approaches (GTAs), which seek to transform harmful gender norms and power imbalances to promote more gender equitability (World Health Organization, 2011), have been successfully carried out (Stewart et al., 2021) in order to address harmful gender norms and practices (Levy et al., 2020). The benefits of successful GTA programs (Levy et al., 2020) include reductions in gender-based violence (Gupta and Sandhya, 2020) and improvements in sexual and reproductive health (SRH) outcomes such as family planning use and contraceptive use (Dagadu et al., 2022).

In Nigeria, evidence shows that gender inequality and social norms that favor men over women remain a major concern. Women are often portrayed as inherently inferior to men, leading to the establishment of rigid gender roles. This arbitrary social construction places men at the forefront, dominating economic and political spheres, while women are confined to domestic roles, primarily engaged in tasks considered menial and lacking in economic and political empowerment (Ajala, 2016).

In 2018, 13.2% of women aged 15–49 years reported that they had been subjected to physical and/or sexual violence by a current or former sexual partner (Nigeria Demographic Health Survey Report, 2018). Moreover, women of reproductive age (15–49 years) often face barriers to their SRH and rights: despite progress, in 2018, only 35.6% of women had their need for family planning satisfied with modern methods (UN Women, 2021).

To address these gender norm issues, and to improve the health and wellbeing of young people, it is necessary to understand the different beliefs of household heads about gender equity and the rights and privileges of young people.

The household is key in society and a head is usually responsible for the household but this is not necessarily the oldest member of the household and may be a male or a female. However, Nigerian household heads are usually male, with more female household heads seen in urban areas (NPC and ICF, 2019).

In developing nations such as Nigeria, there is a widely held belief that the gender of the household head exerts a significant influence on the decision-making processes within the household, including preferences for certain individuals and behaviors. This influence extends to favoring sons over daughters (Morakinyo et al., 2015; Sandström and Vikström, 2015).

Hence, household heads need to be actively engaged in interventions that positively transform negative beliefs on gender equity to improve overall SRH outcomes of adults and young people.

Gender norms are strongly internalized by young people as they grow up within their households and society at large to become men and women (Blum et al., 2017) and this represents a gap of opportunity to promote gender-equitable attitudes before such attitudes solidify, in turn contributing to adverse gender inequality in society. Most studies on household heads focus mainly on female-headed households, household heads’ choice of social amenities, and the social and economic challenges of household heads (Nwaka et al., 2016, 2020; Aryal et al., 2019).

However, there is a paucity of knowledge on the attitude of household heads toward young boys and young girls on the issue of gender norms since the household heads ultimately are responsible for the upbringing of young boys and girls.

This study aimed to assess the gender norm attitudes of male and female heads of households regarding the rights, privileges, and equity promotion of young boys and young girls and also identify factors associated with the gender norm attitudes of household heads toward young people within the household.

The study hypothesis is that socio-demographic characteristics influence the attitudes of male and female heads of households toward the rights, privileges, and equity promotion of young boys and girls, with factors such as age, education level, religious affiliation, and wealth index playing significant roles.

## Method

### Study population

The study population consisted of household heads in households with young people aged 15–24 years, with a mean age of 45.50 years and a standard deviation of 11.14. The household heads were selected from six communities using a modified cluster sampling procedure. Our definition of a cluster was a community governed by a traditional ruler. A sample size of 605 households, 285 male individuals, and 320 female individuals was computed using the guidelines outlined in the Demographic and Health Survey (DHS) Listing Manual 2012 (ICF International, 2012).

To obtain the 605 households, 101 households were systematically drawn on a cluster basis from each of the six purposively selected LGAs with PHCs that provide youth-friendly sexual reproductive services serving as clusters. The participants were recruited until the desired sample size was reached.

### Study design and study area

This study was cross-sectional quantitative research undertaken in six local government areas (LGAs) of Ebonyi State, Nigeria. With an annual growth rate of 2.8%, Ebonyi State has an estimated total population of 4,339,136 and over 355,000 are young people aged 15–24 years (Ebonyi-National Youth Baseline Survey, 2014; Ebonyi State Government, 2023). The six LGAs with the poorest SRH outcomes among young people were purposively selected from the three senatorial zones. These LGAs have a total of 84 primary healthcare centers (PHCs) that provide youth-friendly SRH services and have been prioritized by the state government and partners for scaling up SRH interventions. A community was selected from each LGA based on the stakeholders’ recommendations.

### Study tool and data collection

The data collection instrument was adapted from an annual publication on gender, chapter 5, page 17, which consists of gender norm attitude scales (Nanda, 2011). It was pre-tested in a contiguous state. Research assistants were recruited and trained for 4 days to assist with the data collection. The data were collected for 15 days using paper and electronic copies of the questionnaire. Individual matching of information in the paper and electronic copies of each questionnaire was carried out before the data were uploaded to the server.

### Ethical approval

The protocol for the project leading to the result presented in this study was submitted to the Research and Ethics Committee of Ebonyi State Ministry of Health, with reference number: EBSHREC/07/03/2022-06/02/2026 and, the Health Research Ethics Committee of the University of Nigeria Teaching Hospital Enugu, with reference number: NHREC/05/01/2008B-FWA00002458-IRB00002323. Ethical approval was secured from both committees before community entry and mobilization. Written informed consent was obtained from respondents. Participation was voluntary, and confidentiality was assured.

### Data analysis

In order to examine gender norms and attitudes toward the rights and privileges of men and women and promote girl equity across male and female heads of households, we performed descriptive, bivariate, and logistic regression analyses using Stata.

The regression model allowed us to further the analysis by isolating determinants of gender norms and attitudes on the rights and privileges of young men and women and promoting girl equity across male and female heads of household. Considering variations in individual demographic factors under a regression framework. However, the multivariate regression model can be specified parsimoniously in equation 1 as:


(1) $$Y_{i}=\beta_{0}+\beta_{1}X_{i}+\mu_{i}$$

To generate an attitude score for maintaining the rights and privileges of men and women and promoting girl equity across male and female heads of households, responses were given weighted scores; “1” for a correct response and “0” for incorrect responses.

Thus, we assigned the value of ‘1’ if an individual answered, ‘do not agree’ to a negative statement or ‘agree’ to a positive statement. Also, the value of ‘0’if an individual answered, ‘agree’ to a negative statement or ‘disagree’ to a positive statement. Those who agree to a positive statement (or do not agree with the negative statements) on the rights and privileges of men and women and promoting girl equity across male and female heads of households were judged to have positive attitudes, while respondents who agreed to a negative statement (or do not agree to a positive statement) were judged to have negative attitudes. The total score was converted to a percentage score and used to categorize attitude; scores ≥50% were considered positive, whereas scores below 50% were considered negative.

Based on percentage scores, the outcome variable for individual $\;Y_{i}$ is a dummy variable that takes the value of “1” if an individual score is ≥50% and a value of “0” if an individual score is below 50%. The attitude toward the rights and privileges of men was assessed using six variables. A total of two variables were used to assess the attitude toward the rights and privileges of women and four variables were used to assess the attitude toward promoting girl equity across male and female heads of household.

The *X_i_* is a vector of control variables for individual *i*, which includes (i) gender, (ii) age group, (iii) highest level of education, (iv) marital status, (v) religious affiliation, and (vi) wealth index The logistic regression equation was equated to “1” if gender is female or “0” if gender is male. The error term, $\mu_{i}$ , is taken to be normally distributed. The level of statistical significance was determined by a *p*-value of <0.05.

The household wealth index was calculated using *per capita* household characteristics and asset ownership. The *per capita* household characteristics and asset ownership were used to classify households into socioeconomic quintiles, Q1 to Q4, where Q1 refers to the poorest households and Q4 refers to the richest households.

## Results

The findings showed that the respondents were 52.89% women and 47.11% men (Table 1). The heads of households with an age group 50 years and below were 71.24%, while the heads of households with an age group 50 years and above were 28.76%, with a mean and standard deviation of 45.50 years and 11.14, respectively.

**Table 1:** Socio-demographic characteristics of heads of households.

Variable (*N* = 605)	Frequency (f)	Percent
Gender		
Male	285	47.11
Female	320	52.89
Age-group		
Group 1 (50 years and below)	431	71.24
Group 5 (51 years and above)	174	28.76
Mean (standard deviation) 45.50 years (11.14)		
Highest level of education		
Completed secondary	262	43.31
Completed primary	199	32.89
Completed tertiary	79	13.06
No formal education	65	10.74
Religious affiliation		
Christian–Roman Catholic	284	47.10
Christian–Protestant	282	46.77
Others^a^	37	6.14
Wealth index		
Q1(poorest)	32	5.29
Q2	81	13.39
Q3	274	45.29
Q4 (richest)	218	36.03

a African tradition, Muslim.

Approximately 83.51% of male heads of households significantly (*p*-value = 0.05) disagreed with the statement “the most important reason that sons should be more educated than daughters, is so that they can better look after their parents when they are older” (Table 2).

**Table 2:** Gender norm attitude on rights and privileges of young boys among male and female heads of households.

Variables	Male		Female		*p*-value
	Agree	Disagree	Agree	Disagree	
	F(%)	F(%)	F(%)	F(%)	
Rights and privileges of young boys					
It is important that sons have more education than daughters.	53(18.60)	232(81.40)	62(19.38)	258(80.63)	0.81
The most important reason that sons should be more educated than daughters is so that they can better look after their parents when they are older.	47(16.49)	238(83.51)	35(10.94)	285(89.06)	0.05
If there is a limited amount of money to pay for tuition, it should be spent on the sons first	69(24.21)	216(75.79)	64(20.00)	256(80.00)	0.21
The only thing a woman can really rely on in her old age is her sons.	40(14.04)	245(85.96)	33(10.31)	287(89.69)	0.16
When it is a question of children’s health, it is best to do whatever the father wants.	95(33.33)	190(66.67)	93(29.06)	227(70.94)	0.26

Statistical significance: **p < 0.01, *p < 0.05.

Approximately 62.81% of female heads of household significantly (*p*-value = 0.02) disagreed with the statement “A good woman never questions her husband’s opinions, even if she is not sure she agrees with them” (Table 3).

**Table 3:** Gender norm attitude on rights and privileges of young girls and promotion of equity for girls among male and female heads of households.

Variables	Male		Female		*p*-value
	Agree	Disagree	Agree	Disagree	
	F(%)	F(%)	F(%)	F(%)	
Rights and privileges of young women					
Daughters should be sent to school only if they are not needed to help at home.	22(7.72)	263(92.28)	25(7.81)	295(92.19)	0.97
A good woman never questions her husband’s opinions, even if she is not sure she agrees with them	132(46.32)	132(46.32)	119(37.19)	201(62.81)	0.02*
Promoting equity for girls and women					
Daughters should be able to work outside the home after they have children if they want to.	249(87.37)	36(12.63)	276(86.25)	44(13.75)	0.68
Daughters should have just the same chance to work outside the homes as sons.	240(84.21)	45(15.79)	270(84.38)	50(15.63)	0.96
Daughters should be told that an important reason not to have too many children is so they can work outside the home and earn money.	183(64.21)	102(35.79)	226(70.63)	94(26.38)	0.09
I would like my daughter to be able to work outside the home so she can support herself if necessary.	248(87.02)	37(12.98)	288(90.00)	32(10.00)	1.33(0.25)

Statistical significance: **p < 0.01, *p < 0.05.

Female heads of households aged 50 years and below with an odds ratio of 0.47 (*p*-value = 0.02), suggest that they were 0.47 times more likely to have a positive attitude toward the rights and privileges of young girls (Table 4). Furthermore, female household heads who completed secondary education with an odds ratio of 0.41 (*p*-value = 0.01) indicate that they were 0.41 times more likely to have a positive attitude toward promoting girl equity.

**Table 4:** Logistic regression of attitudes of female heads of household on rights and privileges of young people and promoting girl equity.

Variables	Promoting girl equity			Rights and privileges of young girls			Rights and privileges for young men		
	OR	*p*-value	95% CI	OR	*p*-value	95% CI	OR	*p*-value	95% CI
Age category (50 years and below)	1.06	0.87	0.54–2.08	0.47	0.02*	0.24–0.90	0.66	0.21	0.35–1.26
Level of education complete (primary)									
Completed secondary	0.41	0.01*	0.22–0.77	0.92	0.76	0.53–1.59	1.11	0.70	0.64–1.93
Completed tertiary	0.55	0.17	0.23–1.30	0.87	0.73	0.40–1.91	1.90	0.15	0.80–4.43
No formal education	0.31	0.01*	0.13–0.73	0.44	0.04*	0.20–1.00	0.88	0.75	0.39–1.96
Religious affiliation									
Christian Protestant	0.58	0.42	0.15–2.20	0.27	0.05	0.07–1.00	0.24	0.03*	0.06–0.88
Christian Roman Catholic	0.41	0.19	0.11–1.57	0.41	0.18	0.11–1.50	0.53	0.34	0.14–1.98
Wealth index (poorest)									
Q2	0.27	0.03*	0.08–0.87	1.22	0.69	0.44–3.34	1.51	0.42	0.56–4.12
Q3	0.96	0.94	0.33–2.80	1.02	0.96	0.42–2.48	1.22	0.66	0.51–2.94
Q4 (richest)	0.60	0.37	0.20–1.83	1.09	0.85	0.43–2.76	1.85	0.19	0.73–4.70

Statistical significance: **p < 0.01, *p < 0.05; OR, odds ratio; CL, confidence interval. Variables in the bracket are the groups of interest.

The finding shows that male heads of households who are Christian Roman Catholic with an odds ratio (0.20 *p*-value = 0.04) are 0.20 times more likely to have a positive attitude toward promoting girl equity (Table 5).

**Table 5:** Logistic regression of attitudes of male heads of household on rights and privileges of young people and promoting girl equity.

Variables	Promoting girl equity			Rights and privileges of young girls			Rights and privileges for young boys		
	OR	*p*-value	95% CI	OR	*p*-value	95% CI	OR	*p*-value	95% CI
Age category (50 years and below)	1.94	0.02*	1.14–3.32	1.05	0.84	0.63–1.76	0.87	0.59	0.52–1.45
Level of education complete (primary)									
Completed secondary	0.71	0.26	0.38–1.30	0.77	0.38	0.43–1.38	0.95	0.86	0.53–1.68
Completed tertiary	0.51	0.12	0.22–1.22	0.82	0.65	0.36–1.89	1.19	0.68	0.52–2.77
No formal education	1.54	0.48	0.47–5.05	1.54	0.42	0.54–4.39	1.02	0.97	0.37–2.85
Religious affiliation									
Christian Protestant	0.16	0.02*	0.04–0.65	0.71	0.50	0.26–1.92	0.86	0.77	0.32–2.34
Christian Roman Catholic	0.20	0.04*	0.05–0.86	0.72	0.52	0.27–1.94	0.62	0.35	0.23–1.67
Wealth index (poorest)									
Q2	2.88	0.30	0.39–21.30	2.51	0.44	0.24–26.10	4.60	0.20	0.45–47.45
Q3	3.45	0.19	0.53–22.30	3.85	0.24	0.41–36.27	4.25	0.21	0.45–40.08
Q4 (richest)	2.72	0.30	0.41–18.09	10.46	0.04*	1.08–101.64	8.64	0.06	0.89–83.69

Statistical significance: **p < 0.01, *p < 0.05. OR, odds ratio; CL, confidence interval. Variables in the bracket are the groups of interest.

## Discussion

This study utilized a quantitative research method to assess gender differences in attitudes of heads of households on the rights and privileges of young people and promote girl equity as well. Issues on promoting girl equity, age, and religious affiliation were factors associated with attitudes of male heads of household, while education and wealth index were factors associated with attitudes of female heads of household. Our findings highlight that gender norm attitudes are affected by different factors among male household heads and female ones.

The gender-equitable attitudes seen among female household heads toward the gender norm “that a good woman never questions her husband’s opinions, even if she is not sure she agrees with him” could be influenced by their level of education and career aspiration, as a study carried out on adolescent girls (AGs) revealed that AGs who disagreed with their family member’s belief “that a wife should always obey her husband” were those who had positive education and career aspirations (Closson et al., 2023). In addition, a previous study found protective effects on young people’s health outcomes when a woman can make decisions alone or jointly with her partner (Singh et al., 2015). The reason is that mothers, especially those who give close attention to their children, are typically the first to identify their health outcomes/challenges (Ellis et al., 2013; Dougherty et al., 2020).

Household heads (female and male) disagreed that the most important reason that sons should be more educated than daughters is so that they can better look after their parents when they are older. However, further analysis (regression) showed that gender inequity persists among female and male household heads. Heads of households (female and male) who had formal education and those who had no formal education were more likely to have negative attitudes toward promoting girl equity.

In the context of traditional patriarchal and patriarchal systems, sons are considered to have unique value, as they inherit the family name and property and represent an economic value premium to the family and parents (Sandström and Vikström, 2015; Tilt et al., 2019). This gender norm influences the negative attitude of both educated and uneducated parents toward promoting girl equity. Studies show that parental behavior changes toward a baby as soon as their sex is known or assigned (Mesman and Groeneveld, 2018). Furthermore, boys are consistently encouraged to be strong and independent, whereas girls are seen as vulnerable, ones that should be subordinate, in need of protection, and not be exposed to society (Mesman and Groeneveld, 2018). These gender norms are internalized by parents and they act on it.

A previous study on female heads of households revealed that younger female household heads complained about the challenge of playing multiple roles (head of household and mother role), which put enormous pressure on them and threatened their physical and mental health, causing them to face physical and mental depreciation (Yoosefi Lebni et al., 2020). This physical and mental depreciation could cause one to be aggressive and display harmful gender norms toward young people. This is similar to our findings, which show that female heads of households aged 50 and below have a negative attitude about the rights and privileges of young girls compared to male household heads. Across the world, women juggle work with family and household care responsibilities, and this has a great effect on the attitude of the female head of household toward young people.

Significantly, male heads of households with the highest wealth index (richest) were 10.46 times more likely to have a positive attitude toward the rights and privileges of young girls than female household heads. In developing countries, it is believed that female-headed households are poorer with lower socioeconomic status and are more vulnerable to income shortages than male-headed households (World Bank, 2018; Nwaka et al., 2020). The reasons for this include women’s disadvantaged positions in terms of limited economic opportunities for asset ownership, the family burden associated with unpaid household work, and gender discrimination in the labor market (Nwaka et al., 2016, 2020; World Bank, 2018; Aryal et al., 2019). These reasons contribute greatly to the gender norm attitude of heads of households, in that the one with the greatest economic benefits, is also expected to have greater opportunity/exposure to positive education which in turn reports in the attitude of the individual. The common issue with gender norms is that ideologies/attitudes are passed down from parents to children, and young people growing up in these environments internalize and act on these norms (Kagesten et al., 2016; Dhar et al., 2020; Patel et al., 2021), which then adversely affect their health behaviors and SRH outcomes.

This study is not without some limitations, as there could be information bias attributable to the sensitive nature of the study. However, research assistants were trained to ensure that a conducive environment and neutral attitude with respondents were maintained. Respondents were assured of the confidentiality of the information they provided. The collection of data from a large population would enable the generalizability of the findings. Researchers could utilize a qualitative research method for an in-depth understanding of these gender norms and attitudes seen among male and female household heads.

## Conclusion

Individual and household level characteristics were adversely associated with heads of household attitudes on rights, privileges, and equity promotion for young boys and young girls. The majority of male and female household heads disagreed with the statement “sons need to have a better education than daughters however, female heads of households who have completed tertiary education were less likely tto have a negative attitude toward promoting girl equity.”

There is a need for strategic gender-equitable intervention to address the attitudes of household heads on rights, privileges, and equity promotion for young people across different intersections. To determine similarities and peculiarities, researchers could undertake similar studies in other settings.

## Data availability statement

The raw data supporting the conclusions of this article will be made available by the authors, without undue reservation.

## Ethics statement

The studies involving humans were approved by Research and Ethics Committee of Ebonyi State Ministry of Health, with reference number: EBSHREC/07/03/2022-06/02/2026 and, the Health Research Ethics Committee of University of Nigeria Teaching Hospital Enugu, with reference number: NHREC/05/01/2008B-FWA00002458-IRB00002323. The studies were conducted in accordance with the local legislation and institutional requirements. The participants provided their written informed consent to participate in this study.

## Author contributions

OPN: Writing – original draft. CNE: Writing – review & editing. IA:Writing – review & editing. COM: Writing – review & editing. OO: Writing – review & editing.
